# Association between central obesity and MRI-based biomarkers of knee osteoarthritis independent of BMI: A cross-sectional study from the Osteoarthritis Initiative database

**DOI:** 10.1016/j.ocarto.2026.100810

**Published:** 2026-05-05

**Authors:** Gabby B. Joseph, Fatemeh Dehghani Firouzabadi, John A. Lynch, Zehra Akkaya, Nancy E. Lane, Michael C. Nevitt, Charles E. McCulloch, Thomas M. Link

**Affiliations:** a Department of Radiology and Biomedical Imaging, University of California, San Francisco, USA; b Department of Radiology, Ankara University, Ankara, Turkey; c Department of Rheumatology, University of California, Davis, USA; d Department of Epidemiology and Biostatistics, University of California, San Francisco, USA

**Keywords:** Central adiposity, Knee osteoarthritis, Body mass index, Magnetic resonance imaging, Waist circumference, Waist-to-height ratio

## Abstract

**Objective:**

To evaluate the association between measures of central obesity (waist circumference [WC] and waist-to-height ratio [WHtR]) and MRI-based biomarkers of knee osteoarthritis (KOA), including joint structure, cartilage composition, and synovial inflammation, independent of BMI.

**Design:**

A total of 3186 individuals from the Osteoarthritis Initiative (OAI) cohort with radiographic Kellgren-Lawrence grades 0–3 in the right knee were included. Cartilage composition and joint structural abnormalities were assessed using MRI-based T_2_ cartilage measurements and whole-organ magnetic resonance imaging scores (WORMS). Linear regression was used to investigate the associations between measures of adiposity (WC, WHtR) and MRI-based biomarkers, using standardized predictors and outcomes; the std. β coefficients represent the SD change in outcome per one SD increase in the predictor.

**Results:**

Positive associations between WC and the severity of knee structural abnormalities (WORMS) were found, particularly for cartilage severity scores, even after adjusting for BMI (std. β = 0.093, 95% CI [0.036–0.149], p = 0.001). WC and WHtR were both positively associated with T_2_ values in the lateral femur after adjusting for BMI (WC: std. β = 0.131, 95% CI [0.075–0.186]; WHtR: std. β = 0.193, 95% CI [0.130–0.256]; both p < 0.001). WC and WHtR were associated with MOAKS effusion-synovitis and synovial proliferation before BMI adjustment (all p ≤ 0.001), but not after BMI adjustment (all p ≥ 0.143).

**Conclusions:**

Central obesity is associated with worse cartilage composition and structural abnormalities, independent of BMI. These findings have clinical implications, supporting the role of monitoring and addressing central obesity in the management of knee OA.

## 1 Introduction

Obesity has become a global concern, nearly tripling in prevalence since 1975, with approximately 650 million adults worldwide aged 18 and above being obese, constituting 13% of the global adult population [1]. This condition has been recognized as a risk factor for various diseases, including osteoarthritis (OA), which ranks as the most prevalent joint disease globally [2]. Despite its widespread occurrence and acknowledged genetic influences, the precise mechanism underlying OA remains uncertain [3]. A study by Pottie et al. reported an association between obesity and an accelerated progression of OA, particularly in the knee joint [4]. Another study has shown that chondrocytes degenerate in response to altered mechanical stresses within their microenvironment [5]. Consequently, it has been hypothesized that at least part of the cartilage injury is a result of altered mechanical stress associated with obesity [6]. In addition, certain adipokines, which are cytokines secreted by adipose tissue, are believed to potentially play a significant role in the development of OA through an inflammatory pathway [7]. Thus, both altered mechanical stress (a key factor for cartilage injury) and metabolic factors, driven by inflammatory cytokines and adipokines, play a significant role in accelerating OA development.

Body mass index (BMI) is a commonly used anthropometric measure for classifying obesity; however, it lacks the ability to distinguish between adipose and non-adipose body mass and does not account for variations in fat distribution across the body. This distinction is crucial because, unlike adipose tissue, increased muscle mass (which also contributes to higher BMI) is beneficial for knee OA [8]. Additionally, adiposity in specific regions, such as the abdomen, may have distinct properties with different disease associations [9]. While excessive central adiposity, including both visceral and subcutaneous abdominal fat, has been identified as an independent risk factor for advanced knee OA (such as requiring knee replacement surgery) [10], the relationship between central adiposity and knee OA remains largely unexplored. The waist-to-height ratio (WHtR) has emerged as a superior predictor of various metabolic disorders compared to waist circumference (WC), BMI, or waist-to-hip ratio (WHR) [11]. However, a study by Culvenor et al. suggested that BMI is a stronger predictor of incident knee OA than central adiposity parameters based on data from a small study of 161 cases from the Osteoarthritis Initiative (OAI) database [12]. Understanding which measures of body composition are most closely associated with the development of knee OA holds potential significance in the early clinical management and prognosis of the disease, especially in its pre-radiographic stages.

In this cross-sectional study, our primary objective was to determine whether central adiposity measured by WC and WHtR, independent of BMI, is associated with structural damage in the knee, evaluated semi-quantitatively using MR-based semi-quantitative Whole-Organ Magnetic Resonance Imaging Score (WORMS) and cartilage composition measured with MRI-based T_2_ mapping. Additionally, our secondary aim was to evaluate the relationship between WC and WHtR and knee synovial inflammation using MRI-based measurements of effusion synovitis.

## 2 Method

The Osteoarthritis Initiative (OAI, https://oai.nih.gov) is a multi-center, prospective, longitudinal cohort study that complies with the Health Insurance Portability and Accountability Act. It has received funding from the National Institutes of Health (NIH) and industry partners. The study protocol, any amendments, informed consent documents, and analyses conducted in this study underwent review and approval by the Institutional Review Boards of the OAI clinical centers. Additionally, written informed consent was obtained from all participants involved in the study.

### 2.1 Patient selection

**Fig. 1:**
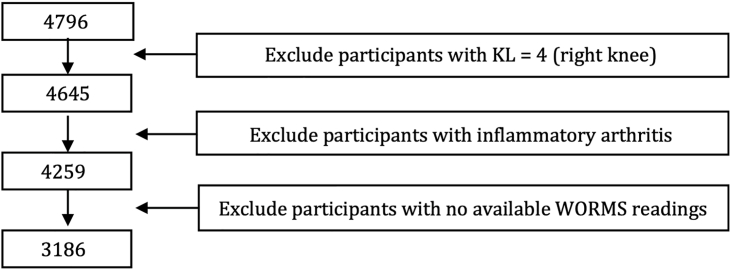
Participant selection from the OAI database. Abbreviations: Kellgren-Lawrence grade (KL), Whole-Organ Magnetic Resonance Imaging Scores (WORMS).

We retrieved data from the publicly accessible OAI database, available at https://nda.nih.gov/oai. The OAI enrolled 4796 participants. In this analysis, inclusion criteria comprised individuals meeting the following criteria: i) radiographic Kellgren-Lawrence (KL) grades 0–3 in the right knee at baseline, and ii) availability of baseline right knee MRIs, including coronal and fat-saturated sagittal turbo spin-echo (TSE) and dual-echo steady-state (DESS) sequences. Exclusions were made for participants: i) with a right knee KL grade of 4 (to focus on non-end-stage disease, where MRI-based structural and compositional measures may better detect OA-related abnormalities), ii) with inflammatory arthritis, and iii) without available Whole-Organ Magnetic Resonance Imaging Score (WORMS) data at baseline. Consequently, a total of 3186 participants met the eligibility criteria in this cross-sectional study; a flowchart illustrating the participant selection is shown in Fig. 1.

### 2.2 Central adiposity and BMI

Central obesity was assessed using the WC and waist circumference-to-height ratio (WHtR), which are widely recognized as a surrogate measure for central adiposity [13]. The WC measurement was obtained at the participant's mid-torso near the umbilicus, which is the area between the lower rib and the iliac crest. A standardized tape measure was used to determine the largest circumference, with measurements taken three times and recorded in centimeters to the nearest 0.1 cm. This method is considered valid for measuring WC and demonstrates high test-retest reliability (ICC = 0.99 for males and ICC = 0.99 for females) as well as intra-rater reliability (ICC = 0.95 for males and ICC = 0.89 for females) [14]. WHtR measurements included an adjustment for body size, dividing WC (cm) by height as recorded in the OAI dataset (mm). BMI (kg/m^2^) was calculated by dividing weight (measured with lightweight clothing only, using a balance beam scale to the nearest 0.1 kg) by height (m) squared [15].

### 2.3 MRI protocol

MRI studies were obtained at four distinct clinical sites within the OAI using 3.0-T scanners that were cross-calibrated (Magnetom Trio, Siemens, Erlangen, Germany) and employed identical quadrature transmit-receive coils (USA Instruments, Aurora, Ohio). To assess structural abnormalities of the right knee semi-quantitatively using WORMS, three sequences were utilized: (a) a coronal 2D intermediate-weighted (IW) TSE sequence (repetition time [TR]/echo time [TE] = 3700/29 ms), (b) a sagittal 3D DESS sequence with water excitation, along with coronal and axial reformations (TR/TE = 16.3/4.7 ms, flip angle = 25°), and (c) a sagittal 2D IW fat-suppressed TSE sequence (TR/TE = 3200/30 ms). Quantitative measurements of cartilage composition based on T_2_ relaxation time were acquired from the right knee using a sagittal 2D multislice multiecho spin-echo (SE) sequence (TR = 2700 ms, TEs = 10, 20, 30, 40, 50, 60, and 70 ms, field of view = 12 cm, slice thickness = 3 mm with 0.5 mm gap, in-plane spatial resolution = 0.31 × 0.45 mm^2^). This sequence was solely conducted on the right knee. Further details regarding the sequences can be found in the OAI MR protocol [16].

### 2.4 MR image analysis

#### 2.4.1 Semi-quantitative MRI scores (WORMS)

Structural abnormalities associated with knee OA were evaluated using a semi-quantitative analysis based on a UCSF-modified WORMS system [17]. Various features were assessed individually: meniscal lesions were graded from 0 to 4; cartilage defects were graded from 0 to 6, and bone marrow edema-like lesions (BML) were graded from 0 to 3 as previously described [17]. The maximum WORMS score for each lesion type was determined as the highest score observed at the knee.

#### 2.4.2 Synovial inflammatory markers

Three sub-features of synovitis were graded for each knee, following protocols outlined in prior publications [[18], [19], [20], [21]]. To address the challenge of distinguishing synovial thickening from intra-articular joint fluid on non-contrast-enhanced MRI (NCE-MRI) using fluid-sensitive sequences, we utilized the term “effusion-synovitis” as previously described [[18], [19], [20], [21]]. Synovitis was assessed using MRI Osteoarthritis Knee Score (MOAKS) effusion-synovitis scores, ACLOAS-based measures and the synovial proliferation scores (SPS) grading, providing a dedicated assessment of synovial inflammation on non-contrast-enhanced MRI and information beyond standard WORMS effusion scores, which only grade percentage distention of the suprapatellar joint space. MOAKS uses axial images to score synovitis and ACLOAS provides a more detailed assessment of the suprapatellar recess. SPS adds information on synovial proliferation within the joint effusion.

The extent of effusion-synovitis was evaluated by measuring the maximum anteroposterior (AP) diameter of the suprapatellar recess in millimeters on a midline sagittal fs IW or DESS image, following the Anterior Cruciate Ligament Osteoarthritis Score (ACLOAS) [21]. Effusion-synovitis was graded from 0 to 3 based on capsular distension, with grade 0 being <2 mm, grade 1 as ≥ 2 to <5 mm, grade 2 as ≥ 5 to <10 mm, and grade 3 as ≥ 10 mm. Second, effusion-synovitis was graded on axial fat-saturated DESS images using the MOAKS, with a 4-point scale: 0 = physiologic fluid; 1 = small amount extending into the retropatellar space; 2 = medium with slight convexity of the suprapatellar bursa; 3 = large with capsular distention. Third, synovial proliferation was assessed on fat-saturated DESS and IW images if the effusion-synovitis score was ≥1 by either ACLOAS or MOAKS, with grades ranging from 1 (smooth synovium) to 3 (extensive thickening with villonodular proliferation) [18,19].

#### 2.4.3 Reproducibility and inter-reader reliability

In a previous study, the intra-class correlation coefficients for the intra-observer agreement were 0.86 (0.801–0.928) for cartilage and 0.87 (0.804–0.932) for meniscus WORMS [17], while the intra-class correlation coefficients for the inter-observer agreement were 0.79 (0.72–0.868) for cartilage and 0.84 (0.771–0.911) for meniscus WORMS. The reproducibility for composite MRI-synovial inflammatory scores was also previously analyzed, and intra-reader agreement values for ACLOAS, MOAKS, and SPS were κ of 0.94, 0.84, and 0.91, respectively. Inter-reader agreement for ACLOAS, MOAKS, and SPS yielded Cohen's κ values of 0.94, 0.90, and 0.86, respectively [17,22,23].

#### 2.4.4 MRI-based cartilage T_2_ relaxation time

Cartilage T_2_ values are quantitative measures of cartilage composition, reflecting water content and collagen matrix integrity, and capturing early, potentially reversible degeneration. Higher T_2_ values indicate increased water content and collagen disorganization, consistent with early degeneration. Mean cartilage T_2_ values were quantified in the entire OAI dataset in five regions: medial and lateral tibia, medial and lateral femur, and patella, using a deep learning-based algorithm [22]. This deep learning-based pipeline, described in detail previously, included reference identification, automated cartilage segmentation across the full dataset, non-rigid morphing guided by cartilage mask extraction, and voxel-wise fitting of the morphed T_2_ images [23].

### 2.5 Statistical analysis

Statistical analysis was performed using STATA version 18 software (StataCorp LP, College Station, TX) with significance set at a two-sided alpha level of 0.05. Linear regression models were used to evaluate the associations between adiposity (WC/WHtR) and primary and secondary outcomes (i.e., primary outcomes: MRI-based biomarkers of knee osteoarthritis (KOA), including WORMS and T_2_ values; secondary outcomes: synovitis scores). These analyses were adjusted for baseline age, sex, and race. In a separate analysis, we additionally adjusted for BMI to determine if the associations were independent of BMI. An additional analysis was conducted with BMI as a predictor, using the same outcomes and adjustments listed above. Standardized predictors and outcomes were defined by subtracting the mean across all participants from each participant's value and dividing by the standard deviation, resulting in variables with a mean of 0 and a standard deviation of 1. The std. β coefficients from the linear regression models represent the SD change in the outcome per one SD increase of the predictor. Given that we conducted multiple analyses across two main exposures (WHtR and WC) and multiple outcomes, we applied a Bonferroni correction to adjust for the increased risk of Type I error due to multiple comparisons. We set statistical significance at a threshold of p < 0.0011, corresponding to the number of comparisons tested.

## 3 Results

### 3.1 Participant characteristics

Participants had a mean age of 60.82 ± 9.12 years and an average BMI of 29.25 ± 4.49 kg/m^2^. Table 1 outlines the baseline distribution of patient characteristics and OA risk factors. The average WC was 103.71 ± 12.33 cm, and the average WHtR was 0.062 ± 0.008. Females comprised 56.6% of the participants, and 79.1% were White or Caucasian. Regarding the KL score, 36.60% had a grade of 0, followed by 30.0% with a grade of 2. Knee Injury and Osteoarthritis Outcome Scores pain and Western Ontario and McMaster Universities Osteoarthritis Index (WOMAC) knee pain scores for participants were 84.42 ± 17.05 and 2.42 ± 3.19, respectively.

**Table 1:** Participant characteristics

	Male	Female	Total
N	1,384 (43.4%)	1,802 (56.6%)	3,186 (100.0%)
Age (years)	60.63 (9.43)	60.98 (8.87)	60.82 (9.12)
WC (cm)	103.91 (11.05)	103.56 (13.23)	103.71 (12.33)
WHtR	0.059 (0.006)	0.064 (0.008)	0.062 (0.008)
BMI (kg/m^2^)	29.10 (3.88)	29.37 (4.91)	29.25 (4.49)
Race			
Other nonwhite	23 (1.7%)	28 (1.6%)	51 (1.6%)
White or Caucasian	1,176 (85.0%)	1,342 (74.5%)	2,518 (79.1%)
Black or African American	179 (12.9%)	415 (23.0%)	594 (18.7%)
Asian	5 (0.4%)	16 (0.9%)	21 (0.7%)
Kellgren-Lawrence scores (right knee)			
Grade 0	549 (39.9%)	608 (34.1%)	1,157 (36.6%)
Grade 1	262 (19.0%)	307 (17.2%)	569 (18.0%)
Grade 2	341 (24.8%)	607 (34.0%)	948 (30.0%)
Grade 3	225 (16.3%)	262 (14.7%)	487 (15.4%)
PASE	177.53 (89.69)	152.41 (77.41)	163.32 (83.88)
KOOS knee pain (right knee)	86.24 (15.53)	83.02 (18.00)	84.42 (17.05)
WOMAC knee pain (right knee)	2.01 (2.79)	2.74 (3.43)	2.42 (3.19)

**Notes:** continuous variables are presented as mean ± standard deviation; categorical variables are shown as number (%). Height was recorded in millimeters in the OAI dataset, and WHtR was calculated accordingly. **Abbreviations:** waist circumference (WC), body mass index (BMI), Western Ontario and McMaster's Osteoarthritis Index (WOMAC), Knee Injury and Osteoarthritis Outcome Score (KOOS), waist-to-height-ratio (WHtR), physical activity score of the elderly (PASE).

### 3.2 Structural knee abnormalities

As shown in Table 2, there was a significant positive association between central obesity (WC) and the severity of WORMS maximum scores of the cartilage (WC std. β = 0.149 [95% CI: 0.116–0.182], p < 0.001) and BMLs (WC std. β = 0.100 [95% CI: 0.066–0.134], p < 0.001). Moreover, cartilage WORMS maximum scores remained significant (WC std. β = 0.093 [95% CI: 0.036–0.149], p = 0.001), even after adjusting for BMI. However, after adjusting for BMI, the association between central obesity (via WC) and WORMS maximum score for BMLs was no longer significant (WC std. β = 0.033 [95% CI: −0.025–0.091], p = 0.262). Also, there was a positive association between central obesity adjusted for height (WHtR) and the severity of WORMS maximum scores in cartilage and BMLs, but the association between central obesity (via WHtR) and WORMS maximum scores for cartilage and BMLs was no longer significant after adjusting for BMI.

**Table 2:** Relationship between obesity measures and WORMS maximum scores.

	Meniscus maximum score			Cartilage maximum score			BML maximum score		
	std. β	95% CI	p-value	std. β	95% CI	p-value	std. β	95% CI	p-value
**BMI-M1**	0.018	(-0.016–0.052)	0.303	0.147	(0.113–0.180)	<**0.001**	0.111	(0.077–0.146)	<**0.001**
**WC-M1**	0.034	(0.000–0.068)	0.048	0.149	(0.116–0.182)	<**0.001**	0.100	(0.066–0.134)	<**0.001**
**WC-M2**	0.057	(-0.001–0.114)	0.052	0.093	(0.036–0.149)	**0.001**	0.033	(-0.025–0.091)	0.262
**WHtR-M1**	0.015	(-0.021–0.051)	0.415	0.130	(0.095–0.166)	<**0.001**	0.086	(0.050–0.123)	<**0.001**
**WHtR-M2**	−0.010	(-0.075–0.055)	0.761	0.002	(-0.062–0.066)	0.957	−0.040	(-0.105–0.026)	0.233

M1: Adjusted Model 1 (age, sex, race)

M2: Adjusted Model 2 (age, sex, race, BMI)

**Notes:** The std. β coefficients from the linear regression models represent the SD change in the outcome per one SD increase in WC/BMI/WHtR. A Bonferroni correction was applied to adjust for multiple testing, setting the statistical significance threshold at p < 0.0011. **Abbreviations**: body mass index (BMI), waist circumference (WC), and waist-to-height-ratio (WHtR), bone marrow edema-like lesion (BML).

There was a positive association between central obesity (via WC) and WORMS regional meniscus scores (lateral body, β = 0.051 [95% CI: 0.016–0.085], p = 0.004). The following regional scores remained significant even after adjusting for BMI: WORMS regional meniscus score (lateral body, β = 0.101 [95% CI: 0.042–0.159], p = 0.001), and regional cartilage scores (lateral femur, β = 0.109 [95% CI: 0.050–0.168], p < 0.001 and lateral tibia, β = 0.174 [95% CI: 0.115–0.233], p < 0.001). Also, there was a positive association between central obesity (via WC) and regional BML scores (lateral tibia, β = 0.100 [95% CI: 0.041–0.159], p = 0.001), even after adjusting for BMI.

### 3.3 Cartilage T_2_

As shown in Table 3, BMI and central obesity (via WC) were significantly and positively associated with T_2_ values (averaged across all compartments, including medial tibia and femur, and lateral tibia and femur) after adjusting for age, sex, and race. Even after adjusting for BMI, significant associations between WC and cartilage T_2_ of the lateral femur were found (WC std. β = 0.131 [95% CI: 0.075–0.186], p < 0.001, Table 3). Additionally, BMI and WHtR were significantly and positively associated with T_2_ values after adjusting for age, sex, and race. After adjusting for BMI, significant associations between WHtR and lateral femur T_2_ values were still found (WHtR std. β = 0.193 [95% CI: 0.130–0.256], p < 0.001).

**Table 3:** Relationship between obesity measures and MRI-based cartilage T_2_ outcomes.

	T_2_ Average of all regions			T_2_ lateral tibia			T_2_ lateral femur			T_2_ medial femur			T_2_ medial tibia		
	std. β	95% CI	p-value	std. β	95% CI	p-value	std. β	95% CI	p-value	std. β	95% CI	p-value	std. β	95% CI	p-value
**BMI-M1**	0.119	(0.085–0.153)	<**0.001**	0.041	(0.006–0.075)	0.022	0.129	(0.095–0.162)	<**0.001**	0.113	(0.080–0.147)	<**0.001**	0.138	(0.103–0.172)	<**0.001**
**WC-M1**	0.113	(0.079–0.146)	<**0.001**	0.059	(0.025–0.094)	**0.001**	0.148	(0.115–0.181)	<**0.001**	0.102	(0.069–0.135)	<**0.001**	0.102	(0.068–0.136)	<**0.001**
**WC-M2**	0.051	(-0.005–0.107)	0.076	0.077	(0.019–0.135)	0.009	0.131	(0.075–0.186)	<**0.001**	0.034	(-0.022–0.090)	0.234	-0.021	(-0.079–0.035)	0.456
**WHtR-M1**	0.134	(0.099–0.170)	<**0.001**	0.070	(0.034–0.107)	<**0.001**	0.171	(0.136–0.206)	<**0.001**	0.120	(0.085–0.156)	<**0.001**	0.106	(0.070–0.142)	**<0.001**
**WHtR-M2**	0.097	(0.033–0.161)	0.003	0.104	(0.038–0.170)	0.002	0.193	(0.130–0.256)	<**0.001**	0.070	(0.006–0.133)	0.032	−0.046	(-0.111–0.019)	0.162

M1: Adjusted Model 1 (age, sex, race)

M2: Adjusted Model 2 (age, sex, race, BMI)

**Notes:** The std. β coefficients from the linear regression models represent the SD change in the outcome per one SD increase in WC/BMI/WHtR. A Bonferroni correction was applied to adjust for multiple testing, setting the statistical significance threshold at p < 0.0011. **Abbreviations:** body mass index (BMI), waist circumference (WC), waist-to-height-ratio (WHtR).

### 3.4 Synovitis

Central obesity (via WC and WHtR) and obesity (via BMI) were found to be significantly and positively associated with both effusion-synovitis scores, using the MOAKS method (WC std. β = 0.108 [0.068–0.148], p < 0.001, and WHtR std. β = 0.088 [95% CI: 0.044–0.131] p < 0.001, respectively), and SPS (WC std. β = 0.089 [95% CI: 0.049–0.129], p < 0.001, and WHtR std. β = 0.070 [95% CI: 0.027–0.114], p = 0.001 respectively) when analyzed separately, after adjustment for age, sex, and race (Table 4). However, after adjusting for BMI, the significant association between central obesity, measured by WC and WHtR, and the MOAKS effusion-synovitis score and SPS no longer persisted (p > 0.05).

**Table 4:** Relationship (std. β and 95% confidence interval) between obesity measures and MRI-synovial inflammatory biomarkers.

	Synovitis-Effusion						Synovial proliferation score		
	ACLOAS			MOAKS					
	std. β	95% CI	p-value	std. β	95% CI	p-value	std. β	95% CI	p-value
**BMI-M1**	0.053	(0.012–0.095)	0.012	0.121	(0.079–0.162)	<**0.001**	0.106	(0.065–0.148)	<**0.001**
**WC-M1**	0.034	(-0.007–0.074)	0.101	0.108	(0.068–0.148)	<**0.001**	0.089	(0.049–0.129)	<**0.001**
**WC-M2**	−0.018	(-0.084–0.047)	0.578	0.042	(-0.023–0.106)	0.210	0.022	(-0.043–0.087)	0.502
**WHtR-M1**	0.014	(-0.029–0.057)	0.521	0.088	(0.044–0.131)	<**0.001**	0.070	(0.027–0.114)	**0.001**
**WHtR-M2**	−0.104	(-0.178– −0.031)	0.005	−0.047	(-0.120–0.026)	0.210	−0.055	(-0.128–0.019)	0.143

M1: Adjusted Model 1 (age, sex, race)

M2: Adjusted Model 2 (age, sex, race, BMI)

**Notes:** The std. β coefficients from the linear regression models represent the SD change in the outcome per one SD increase in WC/BMI/WHtR. A Bonferroni correction was applied to adjust for multiple testing, setting the statistical significance threshold at p < 0.0011. **Abbreviations:** Anterior Cruciate Ligament OsteoArthritis Score (ACLOAS), MRI Osteoarthritis Knee Score (MOAKS), waist circumference (WC), body mass index (BMI), waist-to-height-ratio (WHtR).

## 4 Discussion

In this study, WC was positively associated with the severity of cartilage WORMS maximum scores, even after adjusting for BMI. WC and WHtR were also associated with cartilage T_2_ in the lateral femur after BMI adjustment. These findings likely represent the distinct aspects of cartilage pathology captured by each measure: cartilage T_2_ reflects early compositional changes such as water content and collagen organization, while WORMS captures more advanced structural damage. Together, these results suggest that central obesity is associated with structural knee cartilage degeneration and worse cartilage composition.

In recent years, abdominal obesity has been increasingly recognized as an important risk factor for type 2 diabetes, cardiovascular disease, and other cardiometabolic conditions. However, BMI does not distinguish between abdominal and peripheral fat as well as muscle and bone mass, despite its widespread use in assessing obesity [25,26]. Consequently, the World Health Organization has promoted research on abdominal obesity indices such as WC and WHR to address the limitations of BMI [24]. Our study examined the relationships between general obesity (measured by BMI) and abdominal obesity (measured by WC and WHtR) with MRI-based biomarkers of KOA, providing a framework for evaluating associations between obesity measures and OA-related outcomes. A cohort study by Badley et al. found that both BMI and WHR are strongly associated with obesity and OA, as indicated in a self-reported OA database [25]. Another study reported that being overweight early in adulthood increased the risk of knee and hip OA, based on BMI, WC, hip circumference (HC), and WHR [26]. Christiansen et al. reported that higher WC increased the risk of low physical function in OA patients [27]. Similarly, research showed that elevated WC was associated with a slightly higher risk of disability over time in OA patients [28]. Moreover, Culvenor et al. [12] assessed the relationship between specific local adiposity measures and the risk of incident radiographic KOA, concluding that neither central nor peripheral adiposity was more strongly associated with radiographic knee osteoarthritis (KOA) than BMI. However, their study included only 161 cases and differed from ours in that we used MRI-based biomarkers in a much larger OAI cohort [12]. Taken together, our findings expand on this literature by suggesting that abdominal obesity measures are associated with MRI-based structural and compositional markers of KOA beyond what is captured by BMI alone.

Obesity is a well-established risk factor for OA in the knee and hip, driven by both mechanical loading and systemic inflammation. Various body weight measures, including BMI, fat percentage, and abdominal obesity, are associated with the development of hand OA [29]. In individuals with obesity, OA is characterized by distinct pathological changes, including cartilage degradation, synovial inflammation, and microvascular disruption [30]. These processes may be related to mechanical stress, and inflammatory cytokines are involved in pathways related to hypertrophic chondrocytes, which in turn impact cartilage and subchondral bone [31]. Additionally, obesity-related bone marrow edema-like lesions also contribute to knee OA pathogenesis [31]. Collectively, these findings highlight the multifactorial role of obesity in OA pathogenesis, affecting both biomechanical and biochemical pathways across weight-bearing and non-weight-bearing joints.

Inflammation is a critical factor in the obesity-OA pathway, as adipose tissues produce pro-inflammatory cytokines (e.g., TNF-α, IL-6) and adipokines (e.g., leptin, adiponectin) that regulate chondrocytes [32]. Higher leptin levels are associated with increased cartilage damage, volume loss, and an increased likelihood of requiring knee replacement, making them a potential biomarker for OA prognosis [33]. Adipose tissue inflammation, particularly in the infrapatellar fat pad, exhibits obesity-induced pathologies, including adipocyte hypertrophy and pro-inflammatory macrophage structures, which influence joint load transmission and adipokine production [34]. These inflammatory mechanisms further underscore the active metabolic role of adipose tissue in processes associated with cartilage degeneration and joint deterioration in OA.

MRI transverse relaxation time (T_2_) has been proposed as a biomarker for detecting changes in the composition of articular cartilage. T_2_ reflects collagen integrity, orientation, and hydration, with higher values indicating early cartilage damage. It has been shown to correlate with cartilage histologic grading and mechanical properties [35]. Our findings demonstrated significant associations between central obesity and cartilage T_2_ values in the lateral femur that persisted after adjusting for BMI. Additionally, our results revealed associations between WHtR and average cartilage T_2_, as well as the lateral tibia, lateral femur, and medial femur, which were larger than those between WC and cartilage in the lateral tibia and lateral femur. In previous reports, Gersing et al. demonstrated that a ≥10% total weight loss in individuals with a BMI ≥25 kg/m^2^ slowed the increase of cartilage T_2_ in the medial tibia over 48 months [35]. Another study by Gersing et al. reported that a >5% total weight loss in individuals with a BMI >25 kg/m^2^ significantly reduced T_2_ relaxation times in both medial and lateral knee compartments over 96 months [36]. Interestingly, in patients with Roux-en-Y gastric bypass surgery, it was shown that a lesser amount of total weight loss (<20%) over 12 months was associated with more cartilage T_2_ improvement than a greater amount of weight loss (>20%) [37]. On the other hand, weight loss significantly improved knee pain in both groups, but more so in the group with a weight loss of more than 20%. Thus, central obesity and fat distribution may represent important and potentially modifiable factors associated with early cartilage degeneration, independent of overall BMI and weight loss.

A previous study found an association between synovial proliferation and the maximum scores of menisci and cartilage in WORMS grading [18]. MacFarlane et al. investigated the relationship between cartilage damage progression (defect size and depth) and changes in synovitis effusion over 18 months using MRI to semi-quantitatively grade effusion-synovitis and cartilage damage [38]. They found that 21.3% of the study population had persistent extensive effusion-synovitis over 18 months [38]. Another longitudinal study compared synovitis-effusion volume and score, cartilage volume and defects, and bone marrow lesions at baseline and 24 months using NCE-MRI, finding positive correlations between baseline bone marrow lesions, cartilage defects, osteophytes, joint space narrowing, and changes in effusion-synovitis volume [39]. In our study, central obesity (measured by WC and WHtR) and general obesity (measured by BMI) were significantly and positively associated with the effusion-synovitis score using the MOAKS method and SPS. After adjusting for BMI, the significant association between central obesity (measured by WC and WHtR) and both the effusion-synovitis score using the MOAKS method and SPS no longer persisted. The attenuation of the association between central obesity and synovitis after BMI adjustment suggests this relationship may be driven by overall adiposity rather than central fat distribution alone. Systemic inflammation related to total adipose burden, including circulating adipokines and pro-inflammatory cytokines, is associated with synovial inflammation in OA, and central adiposity measures such as WC and WHtR may not fully capture these effects independently of BMI. A longitudinal study is necessary to further explore this relationship, as the current study employed a cross-sectional design.

A strength of this study is the large sample size and the availability of detailed imaging-based assessments of structural knee pathology. However, several limitations should be noted. One possible explanation for the lack of significant associations in inflammation is that, although inflammation contributes to OA risk, visceral adiposity may not be closely aligned with the most prevalent knee inflammatory phenotypes. Additionally, we did not have measures of specific biologic mediators derived from visceral fat, such as adipokines, that may drive the observed associations. Identifying these active factors will require future longitudinal studies. Potential selection bias due to missing imaging data may affect generalizability; however, baseline characteristics were broadly similar between included and excluded participants, suggesting limited impact on the primary findings. Finally, the complex relationships between overall weight, visceral adiposity, and total body fat composition warrant further investigation.

Overall, our findings suggest that central obesity is independently associated with worsened cartilage composition, as measured by MRI T_2_ relaxation time, and greater structural cartilage degeneration, as assessed by the WORMS grading system. Abdominal fat may offer distinct insights into joint health beyond what is captured by BMI alone, underscoring its potential role in joint degeneration. In this study, WC remained associated with structural cartilage damage after BMI adjustment, while WHtR did not. For cartilage T_2_, both WC and WHtR showed associations in the lateral femur after BMI adjustment. These findings suggest that WC may be more useful for capturing structural cartilage changes independent of BMI, while both WC and WHtR may reflect compositional cartilage changes. While central obesity measures (WC and WHtR) were positively associated with synovitis scores, including the MOAKS-effusion synovitis score method and SPS, these associations were no longer significant after adjusting for BMI. Together, these findings have potential clinical implications, emphasizing the need to monitor and address central obesity as part of a comprehensive approach to managing knee OA. They also highlight the potential for more targeted prevention and treatment strategies that account for the specific risks posed by abdominal fat.

## Contributions

The authors have made substantial contributions to the following sections.

• Conception and design (GBJ FDF JAL ZA NEL MCN CEM TML)
• Analysis and interpretation of the data (GBJ FDF JAL ZA NEL MCN CEM TML)
• Collection and assembly of data (GBJ FDF JAL TML)
• Drafting of the article (GBJ FDF TML)
• Statistical expertise (GBJ CEM)
• Critical revision of the article for important intellectual content (GBJ FDF JAL ZA NEL MCN CEM TML)
• Final approval of the article (GBJ FDF JAL ZA NEL MCN CEM TML)

## Role of funding source

Funding **Source:** This study was funded by NIH R01-AR064771, NIH R01-AR078917, and NIH R01-AG070647. The OAI is a public-private partnership comprised of five contracts (N01-AR-2-2258; N01-AR-2-2259; N01-AR-2-2260; N01-AR-2-2261; N01-AR-2-2262) funded by the National Institutes of Health, a branch of the Department of Health and Human Services, and conducted by the OAI Study Investigators. Private funding partners include Merck Research Laboratories; Novartis Pharmaceuticals Corporation, GlaxoSmithKline; and Pfizer, Inc. Private sector funding for the OAI is managed by the Foundation for the NIH.

## Conflicts of interest

**Disclosure:** All authors declare no competing financial or personal interests.

## References

[1] World Health Organization (2024).

[2] Arden N., Blanco F., Cooper C., Guermazi A., Hayashi D., Hunter D. (2014). Epidemiology of osteoarthritis. Atlas of Osteoarthritis.

[3] Spector T.D., MacGregor A.J. (2004). Risk factors for osteoarthritis: genetics. Osteoarthr. Cartil..

[4] Pottie P., Presle N., Terlain B., Netter P., Mainard D., Berenbaum F. (2006). Obesity and Osteoarthritis: More Complex than Predicted!. Annals of the rheumatic diseases.

[5] Urban J. (1994). The chondrocyte: a cell under pressure. Rheumatology.

[6] Vincent H.K., Heywood K., Connelly J., Hurley R.W. (2012). Obesity and weight loss in the treatment and prevention of osteoarthritis. PM&R.

[7] Gegout P.P., Francin P.-J., Mainard D., Presle N. (2008). Adipokines in osteoarthritis: friends or foes of cartilage homeostasis?. Jt. Bone Spine.

[8] Wang Y., Wluka A.E., English D.R., Teichtahl A.J., Giles G.G., O'Sullivan R. (2007). Body composition and knee cartilage properties in healthy, community-based adults. Ann. Rheum. Dis..

[9] Ferreira I., Snijder M.B., Twisk J.W., van Mechelen W., Kemper H.C., Seidell J.C. (2004). Central fat mass versus peripheral fat and lean mass: opposite (adverse versus favorable) associations with arterial stiffness? The Amsterdam growth and health longitudinal study. J. Clin. Endocrinol. Metabol..

[10] Messier S.P., Beavers D.P., Loeser R.F., Carr J.J., Khajanchi S., Legault C. (2014). Knee-joint loading in knee osteoarthritis: influence of abdominal and thigh fat. Med. Sci. Sports Exerc..

[11] Dong J., Ni Y.-Q., Chu X., Liu Y.-Q., Liu G.-X., Zhao J. (2016). Association between the abdominal obesity anthropometric indicators and metabolic disorders in a Chinese population. Public Health.

[12] Culvenor A.G., Felson D.T., Wirth W., Dannhauer T., Eckstein F. (2018). Is local or central adiposity more strongly associated with incident knee osteoarthritis than the body mass index in men or women?. Osteoarthr. Cartil..

[13] Yoo E.-G. (2016). Waist-to-height ratio as a screening tool for obesity and cardiometabolic risk. Korean journal of pediatrics.

[14] Klein S., Allison D.B., Heymsfield S.B., Kelley D.E., Leibel R.L., Nonas C. (2007). Waist circumference and cardiometabolic risk: a consensus statement from shaping America's health: Association for weight management and obesity prevention; NAASO, the obesity society; the American society for nutrition; and the American diabetes association. Diabetes Care.

[15] Nuttall F.Q. (2015). Body mass index: obesity, BMI, and health: a critical review. Nutr. Today.

[16] Peterfy C.G., Schneider E., Nevitt M. (2008). The osteoarthritis initiative: report on the design rationale for the magnetic resonance imaging protocol for the knee. Osteoarthr. Cartil..

[17] Bucknor M.D., Nardo L., Joseph G.B., Alizai H., Srikhum W., Nevitt M.C. (2015). Association of cartilage degeneration with four year weight gain-3T MRI data from the osteoarthritis initiative. Osteoarthr. Cartil..

[18] Heilmeier U., Mamoto K., Amano K., Eck B., Tanaka M., Bullen J.A. (2020). Infrapatellar fat pad abnormalities are associated with a higher inflammatory synovial fluid cytokine profile in young adults following ACL tear. Osteoarthr. Cartil..

[19] Hunter D.J., Guermazi A., Lo G.H., Grainger A.J., Conaghan P.G., Boudreau R.M. (2011). Evolution of semi-quantitative whole joint assessment of knee OA: MOAKS (MRI osteoarthritis knee score). Osteoarthr. Cartil..

[20] Liu Y., Foreman S.C., Joseph G.B., Neumann J., Tien P.C., Li X. (2019). Is treated HIV infection associated with knee cartilage degeneration and structural changes? A longitudinal study using data from the osteoarthritis initiative. BMC Muscoskelet. Disord..

[21] Roemer F.W., Frobell R., Lohmander L.S., Niu J., Guermazi A. (2014). Anterior cruciate ligament osteoarthritis score (ACLOAS): longitudinal MRI-based whole joint assessment of anterior cruciate ligament injury. Osteoarthr. Cartil..

[22] Razmjoo A., Caliva F., Lee J., Liu F., Joseph G.B., Link T.M. (2021). T2 analysis of the entire osteoarthritis initiative dataset. J. Orthop. Res..

[23] Norman B., Pedoia V., Majumdar S. (2018). Use of 2D U-Net convolutional neural networks for automated cartilage and meniscus segmentation of knee MR imaging data to determine relaxometry and morphometry. Radiology.

[24] Nishida C., Ko G., Kumanyika S. (2010). Body fat distribution and noncommunicable diseases in populations: overview of the 2008 WHO Expert Consultation on Waist Circumference and Waist-Hip ratio. Eur. J. Clin. Nutr..

[25] Badley E.M., Zahid S., Wilfong J.M., Perruccio A.V. (2022). Relationship between body mass index and osteoarthritis for single and multisite osteoarthritis of the hand, hip, or knee: findings from a Canadian longitudinal study on aging. Arthritis Care Res..

[26] Holliday K., McWilliams D., Maciewicz R.A., Muir K., Zhang W., Doherty M. (2011). Lifetime body mass index, other anthropometric measures of obesity and risk of knee or hip osteoarthritis in the GOAL case-control study. Osteoarthr. Cartil..

[27] Christiansen M.B., Thoma L.M., Master H., Voinier D., White D.K. (2020). The association of an increasing waist circumference and risk of incident low physical function in adults with knee osteoarthritis. J. Rheumatol..

[28] Batsis J.A., Zbehlik A.J., Barre L.K., Mackenzie T.A., Bartels S.J. (2014). The impact of waist circumference on function and physical activity in older adults: longitudinal observational data from the osteoarthritis initiative. Nutr. J..

[29] Sobieh B.H., El-Mesallamy H.O., Kassem D.H. (2023). Beyond mechanical loading: the metabolic contribution of obesity in osteoarthritis unveils novel therapeutic targets. Heliyon.

[30] Chen L., Yao F., Wang T., Li G., Chen P., Bulsara M. (2020). Horizontal fissuring at the osteochondral interface: a novel and unique pathological feature in patients with obesity-related osteoarthritis. Ann. Rheum. Dis..

[31] Goldring M.B., Otero M., Plumb D.A., Dragomir C., Favero M., El Hachem K. (2011). Roles of inflammatory and anabolic cytokines in cartilage metabolism: signals and multiple effectors converge upon MMP-13 regulation in osteoarthritis. Eur. Cell. Mater..

[32] Fang H., Judd R.L. (2011). Adiponectin regulation and function. Compr. Physiol..

[33] Martel-Pelletier J., Raynauld J.-P., Dorais M., Abram F., Pelletier J.-P. (2016). The levels of the adipokines adipsin and leptin are associated with knee osteoarthritis progression as assessed by MRI and incidence of total knee replacement in symptomatic osteoarthritis patients: a post hoc analysis. Rheumatology.

[34] Clockaerts S., Bastiaansen-Jenniskens Y.M., Feijt C., De Clerck L., Verhaar J., Zuurmond A.-M. (2012). Cytokine production by infrapatellar fat pad can be stimulated by interleukin 1β and inhibited by peroxisome proliferator activated receptor α agonist. Ann. Rheum. Dis..

[35] Gersing A.S., Solka M., Joseph G.B., Schwaiger B.J., Heilmeier U., Feuerriegel G. (2016). Progression of cartilage degeneration and clinical symptoms in obese and overweight individuals is dependent on the amount of weight loss: 48-month data from the Osteoarthritis Initiative. Osteoarthr. Cartil..

[36] Gersing A.S., Schwaiger B.J., Nevitt M.C., Zarnowski J., Joseph G.B., Feuerriegel G. (2019). Weight loss regimen in obese and overweight individuals is associated with reduced cartilage degeneration: 96-month data from the Osteoarthritis Initiative. Osteoarthr. Cartil..

[37] Lehtovirta S., Kemppainen A., Haapea M., Nevalainen M., Lammentausta E., Kyllönen E. (2024). Effects of bariatric surgery on knee articular cartilage and osteoarthritis Symptoms—A 12-Month Follow-Up using T2 relaxation time and WOMAC osteoarthritis index. J. Magn. Reson. Imag..

[38] MacFarlane L.A., Yang H., Collins J.E., Jarraya M., Guermazi A., Mandl L.A. (2019). Association of changes in effusion-synovitis with progression of cartilage damage over eighteen months in patients with osteoarthritis and meniscal tear. Arthritis Rheumatol..

[39] Wang X., Jin X., Blizzard L., Antony B., Han W., Zhu Z. (2017). Associations between knee effusion-synovitis and joint structural changes in patients with knee osteoarthritis. J. Rheumatol..

